# Telomere Length Correlations among Somatic Tissues in Adult Zebra Finches

**DOI:** 10.1371/journal.pone.0081496

**Published:** 2013-12-09

**Authors:** Sophie Reichert, François Criscuolo, Elodie Verinaud, Sandrine Zahn, Sylvie Massemin

**Affiliations:** 1 Université de Strasbourg, Institut Pluridisciplinaire Hubert Curien, Département Ecologie, Physiologie et Ethologie, Strasbourg, France; 2 Centre national de la recherche scientifique (CNRS), Strasbourg, France; University of Bern, Switzerland

## Abstract

Telomeres are repetitive non coding DNA sequences located at the end of eukaryotic chromosomes, which maintain the integrity of the genome by hiding the chromosome ends from being recognised as double stranded breaks. Telomeres are emerging as biomarkers for ageing and survival, and are susceptible to reflect different individual life history trajectories. In particular, the telomere length with which one starts in life has been shown to be linked with individual life-long survival, suggesting that telomere dynamics can be a proxy for individual fitness and thereby be implicated in evolutionary trade-offs. As a consequence, an increasing number of studies were conducted on telomeres in the fields of ecology and evolutionary biology, in which telomere length was almost exclusively measured from blood samples. However, not only do the number of repeats of the telomeric sequences vary among species, but also within species with great inter-individual telomere lengths variability with age, tissues, and chromosomes. This raises the issue of the exact biological meaning of telomere measurement in blood cells and stimulated the study of the correlation of telomere lengths among tissues over age. By measuring telomere length in adult zebra finches (*Taeniopygia guttata*) in different somatic tissues displaying variable cell turnovers (bone marrow, brain, spleen, pectoral muscle, heart, liver and in red blood cells), we checked that the measure of telomere length in red blood cells is related to telomere lengths in the other tissues. Here we show significant relationships between the telomere lengths of red blood cells and several somatic tissues at adulthood. As red blood cells are easily accessible and suitable for the longitudinal monitoring of the individual rate of telomere loss, our study confirms that telomere length measured in red blood cells could serve as a surrogate for telomere length in the whole avian organism.

## Introduction

Telomeres are repetitive non coding DNA sequences located at the end of eukaryotic chromosomes. They maintain the integrity of the genome by hiding the chromosome ends as being recognised as double stranded breaks by DNA repairing systems [1]. The number of repeats of the telomeric sequences not only varies among species, but also within species with great inter-individual telomere lengths variability with age, tissues, and chromosomes [2].

Telomere length is dynamic and results from pro and anti-erosion factors. Telomere length is progressively lost because of the inability of DNA polymerase to completely replicate telomeres, progressive telomere shortening thus occurs over cell divisions [1]. This loss is thought to be further aggravated by oxidative stress [3] which comes from the imbalance between the production of reactive oxygen species (ROS) and the antioxidant capacity. Telomere length is also regulated by restoration factors such as telomerase activity and the shelterin proteins complex [4], [5]. In the absence of telomere restoration, telomeres get shorter each time a cell divides. Eventually, a lower critical length is reached and telomere signalisation pathway induces cell division arrest and/or cell senescence [1], [6].

Given these characteristics, telomere length and the rates of telomere loss have been widely studied in the context of species or individual variability in lifespan [7]–[12]. The substantial variation observed in the relationship between telomere length and age between individuals [13], and the influence of several environmental factors [14], such as stress [15], [16], on telomere dynamics, hint that telomeres could be more than just an indicator of chronological age [14], [17]–[19]. Indeed, telomeres could be a marker of individual lifestyle [14], [20] and therefore be a good proxy for individual fitness [21]. Consequently, telomeres prove to be of great interest for evolutionary biologists and ecologists as a mechanism potentially involved in the evolution of life histories [14], [16], [20].

In most of the studies conducted on telomeres in the fields of ecology and evolutionary biology, telomere length was measured from blood samples. The main reason is methodological, as blood samples can be easily obtained and repeated during an experiment in most animals, allowing an individual recording of telomere length over time. Indeed, nucleated erythrocytes in birds, reptiles, fish and amphibians, and leukocytes in mammals can be used to provide DNA to measure telomere length from blood samples. As most of the blood cells do not divide once in circulation, telomere lengths in peripheral blood cells are thus susceptible to reflect instantaneous telomere lengths in hematopoietic stem cells [22]. However, the determinants of the rate of telomere dynamics vary greatly between tissue types. For instance, some tissues such as intestinal mucosa and peripheral blood cells have cell rapid turnovers that require high cell proliferation, while other tissues are predominantly mitotically inactive, such as skeletal muscle and the brain. Eroded telomere length can be restored by telomerase activity [23]. Even though telomerase activity is repressed in most normal adult somatic tissues [2], a number of exceptions exist and telomerase activity has been detected in proliferative cells of tissues and organs having rapid cell turnovers [2], [24]. Telomerase is generally active in germ cell lines, in embryonic tissue and in tissues with a high cell turnover such as bone marrow, intestine and gonads [2], [24]. Such inter-tissue differences in the rate of cell division and of telomere anti-erosion activities should lead to large differences in telomere shortening rates [2], [25], with shorter telomeres in highly replicative tissues [26], [27].

This raised the issue of the exact biological meaning of telomere measurement in blood cells and had stimulated the study of the correlation of telomere lengths among tissues over age (i.e. synchrony in telomere length). Data in human studies highlight strong correlations in telomere length across somatic tissues [25], [28], [29]. This synchrony in telomere lengths across somatic tissues was found in other mammals (monkeys [26] or dogs [30]) where leukocytes telomere length was similar to the one measured in other somatic tissues such as muscle, skin and fat. A comparable pattern was found in the zebrafish (*Danio rerio*), characterised by little difference in telomere lengths among organs [31].

However, corresponding data are lacking in other vertebrates species, such as birds. By measuring telomere length in adult zebra finches (*Taeniopygia guttata*) in different somatic tissues with variable cell turnovers such as the bone marrow, the brain, the spleen, the pectoral muscle, the heart, the liver and in red blood cells (RBC), we propose to determine if the measure of telomere length in red blood cells is related to the telomere lengths in these other tissues. We also aim to verify if the rate of telomere loss differ among tissues in different adult age classes.

## Methods

### Ethics statement

During this experiment, animal care was in accordance with institutional guidelines. The study complied with the ‘Principles of Animal Care’ publication no. 86–23, revised 1985 of the National Institute of Health, and with current legislation (L87-848) on animal experimentation in France and with the European Instruction 2010/63/UE of the 22nd September 2010. The DEPE holds a license from the French Department of Veterinary Service (license number G67-482-18). Dr François Criscuolo, team leader of this project, holds a license to experiment on animals delivered by the French prefecture (license number 67–78). Animals were originally obtained from a pet shop (Nilufar Strasbourg Lampertheim, Lampertheim, France).

The euthanasia method complies with the CNRS instructions regarding animal experimentation.

Prior to euthanasia, animals were in cages (0.57×0.31×0.39 m) with food (a commercial mix of seeds for exotic) and water *ad libitum*. The cages were put in a room with constant temperature of 24°C (**±**1°C) and light conditions were 13 L: 11 D.

### Experimental design

The present study was conducted on twenty captive adult zebra finches (*Taeniopygia guttata*) (10 males and 10 females). Before euthanasia, a blood sample was taken (50 µL) and snap-frozen into liquid nitrogen, and birds were then sacrificed by cervical dislocation. The brain, the spleen, the heart, the liver, the right pectoral muscle and the bone marrow were collected just after euthanasia and snap-frozen into liquid nitrogen. Blood and tissue samples were stored at −80°C until analysis. Mean animal age was 3.7 years and ranged from one to seven years old.

### Telomere length measurement

Telomere length was measured on DNA extracted from each tissue, using DNeasy Blood and Tissue kit (Qiagen). Telomere length was assessed by quantitative real-time amplification (qPCR) procedure [32] adapted to zebra finches and described by Criscuolo [33]. Cycle number is proportional to the sample telomere length (T), or to the number of copies of a non-variable copy number gene (or control gene S). Relative telomere length for each sample is expressed as the ratio (T/S). We used glyceraldehyde-3-phosphate dehydrogenase (GAPDH) as a single control gene. Forward and reverse primers for the GAPDH gene were 5′-AACCAGCCAAGTACGATGACAT-3′ and 5′-CCATCAGCAGCAGCCTTCA-3′ respectively. Telomere primers were: Tel1b (5′-CGGTTTGTTTGGGTTTGGGTTTGGGTTTGGGTTTGGGTT-3′) and Tel2b (5′-GGCTTGCCTTACCCTTACCCTTACCCTTACCCTTACCCT-3′). qPCR for both telomere and GAPDH were performed using 5 ng of DNA with sets of primers Tel1b/Tel2b (or GAPDH-F/GAPDH-R), each used at a concentration of 200 nM/200 nM, in a final volume of 10 µl containing 5 µl of Power SYBR Green PCR Master Mix (Applied biosystems). Telomere and GAPDH real time amplifications were performed on separate plates. Samples were run in duplicate on each plate. Samples were randomly assigned on a total of four plates with samples of one or two tissues source per plate. Each plate (telomere and GAPDH) included serial dilutions (40 ng, 20 ng, 10 ng, 5 ng, 2.5 ng, 1.25 ng) of DNA of the same reference bird which were run in duplicate. This was used to generate a reference curve to control for the amplifying efficiency of the qPCR. Mean r^2^ of the qPCR runs were 0.95 for telomere and 0.99 for the control gene. Mean amplification efficiency of the qPCR runs ranged between 105 and 110 for telomere and between 104 and 110 for the control gene. qPCR conditions for telomeres were 10 min at 95°C followed by 50 cycles of 30 s at 94°C, 34 s at 62°C and 30 s at 74°C. qPCR conditions for the GAPDH were 10 min at 95°C followed by 50 cycles of 1 min at 95°C and 1 min at 60°C.

Intra-plate mean coefficients of variation for Ct values were 1.77% for the telomere assay and 0.65% for the control gene assay. Inter-plate coefficients of variation based on repeated samples were 2.8% for the telomere assay and 2.4% for the control gene assay (Ct values again). To take into account the slight variation of efficiencies between telomere and control gene amplifications, we calculate relative telomere length using the method suggested by Pfaffl [34]. The mean values were used to calculate the relative T/S ratios using the formula: ((1+E telomere)^∧^ ΔCt telomere (control – sample)/(1+E control gene)^∧^ ΔCt control gene (control – sample)). Both a negative control (water) and melting curves were run for each plate to control for the absence of (i) non-specific amplification and of (ii) primer-dimer artefact.

### Statistical analysis

Statistical analyses were performed in three steps. First of all, to test whether telomere length differed between tissues, telomere length was analysed using a generalized estimating equations (GEE) model. To account for the fact that tissues were coming from the same individual, the type of tissue (RBC, the brain, the spleen, the heart, the liver, the right pectoral muscle and the bone marrow) was included as a repeated variable (tissues being repeated within individuals) in the model structure. Sex, as well as the interaction between sex and tissue type were added as fixed factors. The potential effect of age on telomere length was controlled for by using individuals' age as a covariate. For the GEE analysis, post hoc comparisons among tissues were conducted using Bonferroni tests.

Secondly, Pearson's correlations were used to test the significance of the relationships between (a) telomere length and age within the different tissues and (b) telomere lengths among the different tissues. Because of an extreme outlier individual presenting long telomere lengths which may drive most of the linear relationship, analyses were also performed without this individual (see results).

Finally, similarly to the analysis conducted in [28], we assessed if the differences in telomere length among RBC and other tissues remained stable over age. To do so, ANOVA was conducted on telomere length differences (i.e. RBC – tissue X), in which Sex, Age and the interaction Sex × Age were used as fixed factors.

Normal distribution (Shapiro-Wilk tests) and equality of variances (Levene tests) were respected for telomere lengths (all p>0.05).

All statistical analyses were performed using SPSS v. 18.0.

## Results

No significant progressive erosion of telomere length with age was detected in any of the tissues studied (Tables 1, 2, 3).

**Table 1:** Determinants of telomere length evaluated in different tissues of adult zebra finches (GEE analysis).

Variable	Wald chi-squared	P
**Telomere length**		
Sex	,958	,328
Tissue type	36,951	**<,001**
Age	,163	,687
Sex*Tissue	10,966	,089

Data were analysed to determine the impact of the type of tissue (repeated variable within individual), sex, and the interaction between sex and the type of tissue (fixed factors). Individuals' age potential impact on telomere length variability was controlled for (covariate). Bold value indicates a significant effect (P≤0.05).

**Table 2:** Results of the Pearson's correlations testing the linear relationships between telomere lengths in different tissues, as well as the relationships between telomere length and age in the different tissues (right column).

		red blood cells	bone marrow		spleen		muscle		heart		liver		brain		age	
red blood cells	r	1	,325	−,*120*	0,789	*0,79*	0,57	,*172*	0,479	,*133*	0,646	*0,67*	0,724	*0,79*	−,144	−,*208*
	p		,174	,*635*	**,000**	**,** ***000***	**,014**	,*509*	**,044**	,*611*	**,032**	**,** ***034***	**,003**	**,** ***001***	,546	,*392*
bone marrow	r		1	*1*	,281	,*281*	0,723	,*184*	,322	−,*418*	0,861	,*250*	0,896	,*166*	,053	,*020*
	p				,275	,*275*	**,000**	,*464*	,179	,*084*	**,000**	,*458*	**,000**	,*571*	,825	,*935*
spleen	r				1	*1*	,259	,*259*	,272	,*272*	0,791	*0,79*	0,638	*0,64*	−,281	−,*281*
	p						,333	,*333*	,308	,*308*	**,006**	**,** ***006***	**,014**	**,** ***014***	,274	,*274*
muscle	r						1	*1*	0,709	,*105*	0,943	,*027*	0,932	,*027*	,165	,*325*
	p								**,001**	,*678*	**,000**	,*938*	**,000**	,*929*	,499	,*188*
heart	r								1	*1*	0,796	−,*027*	0,73	,*099*	,151	,*152*
	p										**,002**	,*937*	**,003**	,*747*	,537	,*547*
liver	r										1	*1*	0,979	,*616*	,005	−,*361*
	p												**,000**	,*141*	,988	,*276*
brain	r												1	*1*	−,044	−,*452*
	p														,876	,*105*
age	r														1	*1*
	p															

Results of the same Pearson's correlation analysis after removing the outliner point (the individual presenting the longest telomeres) from the analysis are presented in italics.

**Table 3:** Result of separate analyses of variance for the difference in red blood cells (RBC) and somatic tissues' telomere length in relation to Sex, Age and the interaction Sex × Age.

Univariate analysis		
*RBC - Muscle*	*F*	*p*
Age	,952	,345
Sex	,714	,411
Age × Sex	1,355	,263
*RBC – Bone marrow*		
Age	,282	,603
Sex	3,123	,098
Age × Sex	2,571	,130
*RBC - Spleen*		
Age	,070	,795
Sex	5,422	,354
Age × Sex	2,945	,108
*RBC – Heart*		
Age	,724	,408
Sex	1,050	,322
Age × Sex	,791	,388
*RBC – Liver*		
Age	,765	,407
Sex	1,596	,242
Age × Sex	2,673	,141
*RBC - Brain*		
Age	,106	,750
Sex	,025	,878
Age × Sex	,128	,727

### Tissue differences in telomere lengths

**Figure 1:**
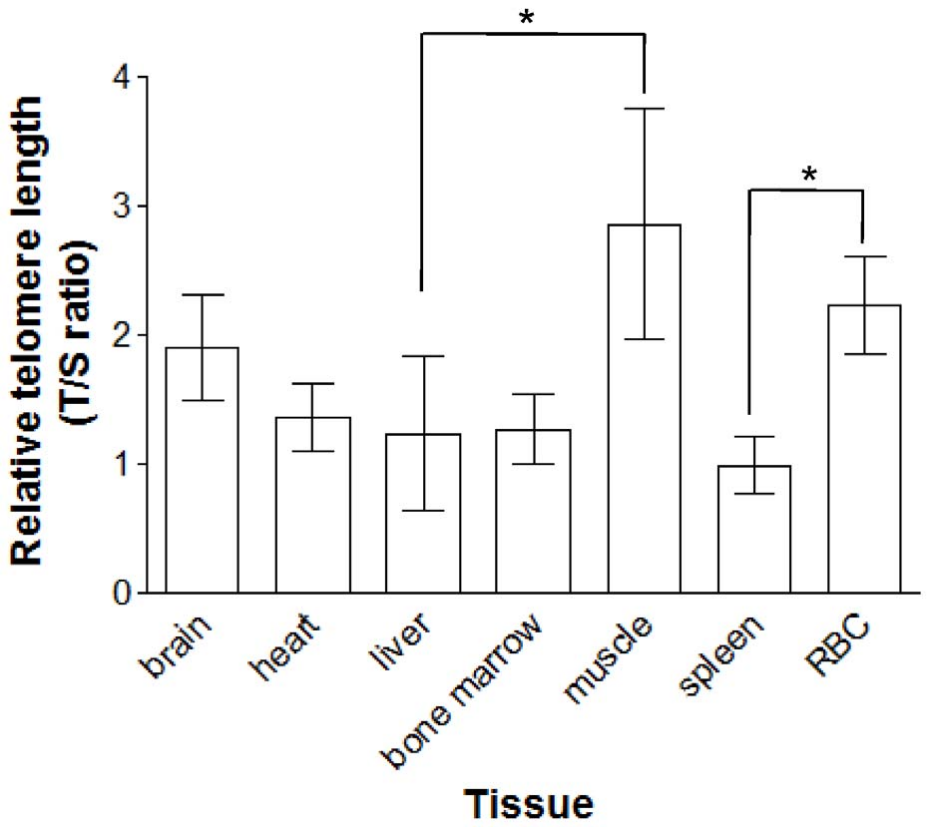
Mean relative telomere lengths (±SE) in the different somatic tissues (brain, heart, liver, bone marrow, muscle, spleen, RBC), tissues presenting significantly different telomere lengths are marked with an asterisk.

The type of tissue had a significant effect on telomere lengths (p<0.001; Table 1). As the sex effect, the interaction between sex and tissue type, and age were not significant (Table 1). Post-hoc analyses revealed that the pectoral muscle was characterised by significantly longer telomere length than liver telomere length (Bonferroni posthoc comparisons p = 0.015), but not significantly so from the other tissues (Bonferroni posthoc comparisons, brain p = 1; heart p = 0.884; bone marrow p = 0.631; spleen p = 0.841 and RBC p = 1) (Figure 1, Table 4). RBC telomere length was not significantly different from telomere lengths in the other tissues (brain p = 1, heart p = 0.303, liver 0.922, bone marrow p = 0.260, pectoral muscle p = 1) except for the spleen (p<0.001). Telomere lengths in the other tissues (brain, heart, liver, bone marrow, and spleen), were not significantly different from the others (Figure 1, Table 4).

**Table 4:** Mean telomere lengths measured by qPCR in different tissues of adult zebra finches. Values are mean ±SE.

	brain	heart	liver	bone marrow	muscle	spleen	red blood cells
Telomere length	1,905	1,368	1,235	1,269	2,859	,991	2,229
	(,403)	(,263)	(,597)	(,267)	(,897)	(,217)	(,377)

**Figure 2:**
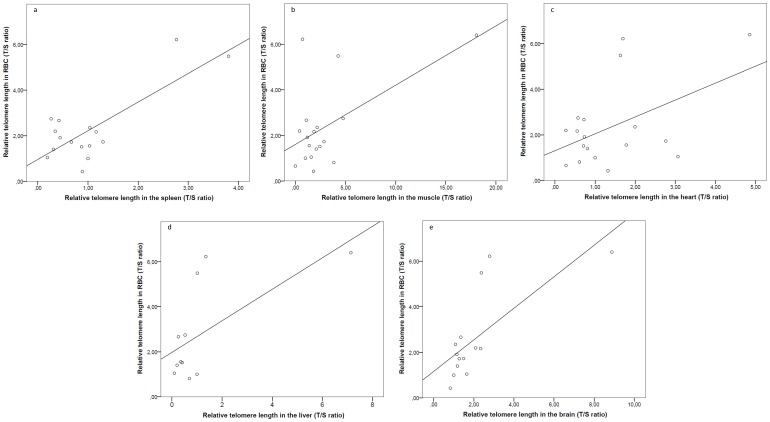
Correlations between red blood cells telomere length and telomere lengths in different somatic tissues. a. Correlation between red blood cells telomere length and spleen telomere length. b. Correlation between red blood cells telomere length and muscle telomere length. c. Correlation between red blood cells telomere length and heart telomere length. d. Correlation between red blood cells telomere length and liver telomere length. e. Correlation between red blood cells telomere length and brain telomere length.

Individual zebra finches showed significant correlations of their telomere lengths measured in RBC and those obtained in the additional tissues studied. Consequently, individuals presenting long RBC telomere lengths were also presenting long telomeres in the spleen (Figure 2a), the muscle (Figure 2b), the heart (Figure 2c), the liver (Figure 2d) and the brain (Figure 2e) (respectively p<0.001, p = 0.014, p = 0.044, p = 0.032, p = 0.003). Surprisingly, the only tissue for which there was a lack of relationship with red blood cells was the bone marrow (p = 0.174) (Table 2). Telomere lengths in the brain, the liver and the muscle were also correlated to telomere lengths in several other somatic tissues (Table 2).

In our data, there was an individual carrying particularly long telomeres. A potential bias may have been induced by the extreme telomere values of this individual. Accordingly, we performed an additional Pearson's correlation analysis without this individual (Table 2, values in italics). Again, no significant link was detected between telomere lengths in the different tissues and age. In addition, RBC telomere lengths were still correlated to telomere lengths in the spleen, the liver and the brain (respectively p<0.001, p = 0.034, p = 0.001) but not to those in the muscle and the heart (respectively p = 0.509, p = 0.611). Telomere lengths in the liver and the brain were still correlated with telomere lengths in other tissues, but it was no longer the case for the muscle any more (Table 2, values in italics).

### Relative rate of telomere loss among tissues

Differences between RBC and other tissues' telomere length were constant with individual age (Table 3), indicating that ageing does not change the relationship among tissue telomeres (other comparisons NS, data not shown).

## Discussion

Telomeres, through their impact on cell senescence [35], are supposed to be part of the multifactor process of ageing in most eukaryotes [6], [14], [36]. Telomere length measured in blood cells has been well correlated with individual lifespan or survival rate in humans or captive and free-ranging animals [7], [8], [10], [11], [17]. For instance, telomere length measured from RBC in captive zebra finches at the end of growth (25 days) was positively related to individual lifespan [11], suggesting that our understanding of ageing processes will pass through our understanding of the telomere-driven ageing process. In this context, determining accurately how telomere dynamics in peripheral blood cells relates to telomere lengths in the whole organism is an important starting question.

By finding significant relationships between telomere length of RBC and telomere lengths of several other somatic tissues independently of age, our study confirms that telomere length measured in RBC could serve as a surrogate for telomere length in the whole avian organism.

However, we ought to be cautious with these findings. Indeed, when the individual carrying the longest telomeres was removed from the analysis, the relationships between RBC and other somatic tissues telomere lengths remained true only for the spleen, the liver and the brain. This actually reduces the weight of the conclusions that may be driven from our data, because of the relative small sample size. However, rather than limiting the interest in using RBC telomeres as an ageing proxy for the whole organism, it suggests that further data needs to be collected in the future to assess why certain tissues, such as the skeletal muscle or the heart, may present particular telomere erosion rates.

*In vitro* experiments have actually demonstrated that telomere length is decreasing while cells divide [35]. As a consequence, highly replicative tissues should present shorter telomeres on average than post-mitotic ones. However, since the discovery of the enzyme telomerase and of its “immortalization activity” *via* telomere length maintenance [23], we know that erosion of telomeres, with time, may be counter-balanced [37]. This is actually the case early in development (i.e. when telomerase is highly active in a large number of tissues, for birds see [24]) and thereafter in specific tissues, particularly those containing a large pool of stem cells ([2], [24]). As a consequence, telomere loss is likely to vary among tissues depending on their replicative potential and/or their capacity to retain telomerase activity [38] with telomere length resulting from the impact of these two factors. Our data seem to fit with those predictions. The spleen, a highly replicative tissue, which displays a high telomerase activity at adulthood ([2], [24]), was characterised by shorter telomeres compared to the RBC. Interestingly, the shorter telomeres in the liver compared to muscle telomere length, suggested in our case that this organ of prime metabolic role may face particular pro-ageing challenges (see also long muscle telomeres in mammals; [28], [30]).

Altogether, these results suggest that replicative tissues, with known telomerase activity, may have short telomeres compared to non-replicative tissues. This supports the hypothesis that telomerase *per se* is not the guarantee of an absolute long telomere length. Rather, telomerase should be viewed as a factor that helps to maintain telomere lengths above a threshold value (e.g. in particular short telomeres, see [39]), preserving the renewal capacities of stem cells [40]. Other mechanisms may also be implicated such as particular shelterin protein expression [41]. It also confirms that maintaining a proliferative capacity in a given tissue is done at a potential cost in terms of telomere erosion, probably early in development to set-up a large progenitor cell pool, as recently suggested [28], [42].

Our study shows that in adult zebra finches, RBC telomere length is correlated to telomere lengths in other somatic tissues independently of their replicative status. Similar relationships have been found in humans (using leucocytes, [25], [28]). Telomere lengths in peripheral blood cells are susceptible to reflect telomere lengths in hematopoietic stem cells as was indicated by previous studies [22]. However, in our case there was no correlation of RBC telomere length with telomere length in bone marrow cells. A first explanation may be that we extracted different cell types in our bone marrow samples, potentially blurring our telomere measurement with osteoclasts/osteoblasts family types. Additionally, given our relatively small sample size (n = 20), confounding factors such as age or sex that, even if they were taken into account in our model, may have led to the non-significant relationship. The study of the telomerase activity in avian RBC or particular expression of shelterin proteins may be of prime interest to tackle this question.

Importantly, the differences among RBC and the different somatic tissues studied were kept stable with age in our bird model, indicating that rates of telomere loss may not be significantly different among tissues at adulthood. This supports the idea that differences among tissues in telomere length are probably set-up during the growth period (pre- and post-hatch/birth;[11], [13], [38], [43]) and that it is fixed thereafter during adult life [28]. However, we still lack information on the nature of the cellular mechanisms that may explain why proliferative and non-proliferative tissues are losing telomeres at the same rate in adult zebra finches. It may be valuable to look at tissues' telomere attrition in species known to keep telomerase activity at adulthood [24]. In addition, we were not able to establish that telomere length was a function of age, probably because of the survival of the best individuals at old ages (i.e. those having the longest telomeres). Such an absence of significant telomere shortening in non-replicative tissue has been previously described, probably for similar reasons as in [30].

Our study gives support of telomere length synchrony in adults only. Telomere dynamics has been found to vary between chicks and adults in different bird species [13], [44]. For this reason, relationships between telomere lengths of somatic tissues might differ in the young age class than the one we observed. The possibility of accelerated telomere shortening in tissues of rapid expansion during growth compared to late-developing ones has indeed been proposed in a recent study conducted on humans [28]. Consequently we still need to look at telomere synchrony among organs in early life in zebra finches (i.e. during the first 30 days of life). Furthermore, in birds, telomere dynamics and telomere length maintenance mechanisms differ between short-lived and long-lives species [10], [44], thus the links between telomere lengths in different somatic tissues might also depend on the longevity of the species considered. Telomere length synchrony between organs should then also be tested in long-lived species.

It is still the case that for evolutionary biologists, using RBC's telomere measurement seems to be representative of the whole organism telomere picture, explaining why peripheral blood telomeres has been found as a proxy for adult individual health status [8], [45] and survival [7], [11], [12].
